# The Bile Acid Sensor FXR Protects against Dyslipidemia and Aortic Plaques Development Induced by the HIV Protease Inhibitor Ritonavir in Mice

**DOI:** 10.1371/journal.pone.0013238

**Published:** 2010-10-08

**Authors:** Andrea Mencarelli, Sabrina Cipriani, Barbara Renga, Daniela Francisci, Giuseppe Palladino, Eleonora Distrutti, Franco Baldelli, Stefano Fiorucci

**Affiliations:** 1 Dipartimento di Medicina Clinica e Sperimentale, University of Perugia, Facoltà di Medicina e Chirurgia, Perugia, Italy; 2 Dipartimento di Medicina Sperimentale e Scienze Biochimiche, University of Perugia, Facoltà di Medicina e Chirurgia, Perugia, Italy; 3 Azienda Ospedaliera di Perugia, Ospedale Santa Maria della Misericordia, Perugia, Italy; Centro Cardiologico Monzino, Italy

## Abstract

**Background:**

Although human immunodeficiency virus (HIV)–related morbidity and mortality rates in patients treated with a combination of high active antiretroviral therapy (HAART) have declined, significant metabolic/vascular adverse effects associated with the long term use of HIV protease inhibitors (PIs) have emerged as a significant side effect. Here we illustrate that targeting the bile acid sensor farnesoid X receptor (FXR) protects against dyslipidemia and vascular injury induced HIV-PIs in rodents.

**Methodology/Principal Findings:**

Administration of the HIV PI ritonavir to wild type mice increased plasma triacylglycerols and cholesterol levels and this effect was exacerbated by dosing ritonavir to mice harbouring a disrupted FXR. Dyslipidemia induced by ritonavir associated with a shift in the liver expression of signature genes, Sterol Regulatory Element-Binding Protein (SREBP)-1 and fatty acid synthase. Treating wild type mice with the FXR agonist (chenodeoxycholic acid, CDCA) protected against development of dyslipidemia induced by ritonavir. Administration of ritonavir to ApoE^−/−^ mice, a strain that develop spontaneously atherosclerosis, increased the extent of aortic plaques without worsening the dyslipidemia. Treating these mice with CDCA reduced the extent of aortic plaques by 70% without changing plasma lipoproteins or the liver expression of signature genes. A beneficial effect on aortic plaques was also obtained by treating ApoE^−/−^ mice with gemfibrozil, a PPARα agonist. FXR activation counter-regulated induction of expression/activity of CD36 caused by HIV-PIs in circulating monocytes and aortic plaques. In macrophages cell lines, CDCA attenuated CD36 induction and uptake of acetylated LDL caused by ritonavir. Natural and synthetic FXR ligands reduced the nuclear translocation of SREBP1c caused by ritonavir.

**Conclusions/Significance:**

Activation of the bile acid sensor FXR protects against dyslipidemia and atherosclerotic caused by ritonavir, a widely used HIV PI. From a mechanistic stand point it appears that besides reducing the liver expression of genes involved in fatty acid synthesis, FXR activation counter-regulates the expression/activity of CD36 on monocytes. FXR ligands might hold promise in the treatment dyslipidemia induced by ritonavir.

## Introduction

Protease inhibitors (PI) as a part of highly active anti-retroviral therapy (HAART) have been used successfully in the treatment of human immunodeficiency virus (HIV) infection. Incorporation of HIV protease inhibitors in the HAART causes profound and sustained suppression of viral replication, significantly reduces the morbidity and mortality, and prolongs the lifespan of patients with HIV infection [1], [2]. HAART has changed the clinical profile of HIV infection from a sub-acute lethal disease to a chronic ambulatory disease [2], [3]. Despite its efficacy in controlling the disease progression, the use of PI therapy associates with an increased risk of development of premature atherosclerosis. This is particularly true in younger patients (men under the age of 34 and women under the age of 44) [1]–[4]. Although the mechanism underlying HIV PI-induced atherosclerosis remains to be fully identified, an increasing body of evidence suggests that ttreatment of HIV-infected patients with HIV PIs causes a dyslipidemia which contributes to the development of cardiovascular diseases [4]. A significant increase in plasma triacylglycerols and total cholesterol concentrations, often associated with abnormal body fat distribution and peripheral insulin resistance (hyperinsulinemia, hyperglycemia and diabetes mellitus), has been detected in HIV PIs-treated patients [5]–[8]. There is evidence that such pro-atherogenetic lipid profile induced by PIs in linked to the effect these agents exert on the Sterol Regulatory Element-Binding Protein (SREBP)-1 and 2. SREBPs are master genes and transcription factors that sense liver cholesterol concentrations. In hepatocytes and adipocytes, activation or extended life span of SREBP proteins, resulting from suppression of activated SREBP degradation, causes the nuclear accumulation of activated SREBP-1 and 2 leading to increased lipogenesis by induction of lipogenetic genes such as the fatty acid synthase (FAS) [9], [10].

In addition to these effects, PIs act directly on macrophages, another important player in the formation of atherosclerotic plaques. In *vitro* studies have shown that exposure of macrophages to HIV PIs increases the accumulation of free cholesterol, activates the unfolded protein response (UPR), induces apoptosis, and promotes transition toward a foam cell phenotype [11]. Macrophage's recruitment into the vascular wall is one of the earliest events in atherogenesis. This event is mediated by the up-regulation of the scavenger receptor-dependent uptake of lipoprotein sterols by macrophages in the sub-endothelial space, which contributes to the formation of lipid-laden macrophages [13]. The class B scavenger receptor, CD36, mediates the uptake of oxidized (ox) LDL particles by macrophages [13] and is a well identified target of HIV PIs, although the mechanisms that regulate the induction of its expression/activity in response HIV PIs remain elusive [12].

FXR (NR1H4) is an adopted member of the nuclear receptor super-family of ligand-activated regulatory factor highly expressed in entero-hepatic tissues. In the liver, FXR serves as a bile acid sensor and regulates bile acid synthesis and excretion [14]. In target tissues, primary bile acids, cheno-deoxycholic acid (CDCA) and cholic acid (CA), bind and activate the receptor at micromolar concentrations leading to feedback repression of bile acid biosynthesis, an effect that is primarily achieved by repressing the expression/activity of the cholesterol 7α-hydroxylase (CYP7A1), a gene encoding for a protein that regulates the first, and rate limiting, enzymatic step of cholesterol breakdown. Other than bile acid synthesis, FXR intervenes in a variety of physiological processes. In mice, FXR deficiency results in a pro-atherogenic serum lipoprotein profile [15], while FXR agonism reduces triacylglycerols by repressing the expression and activity of SREBP1c in the liver. The phenotypic readouts of these effects is a reduction of the pro-atherogenic lipid profile and an attenuation of the tendency toward development of atherosclerotic plaques in ApoE^−/−^ and LDLr^−/−^ mice [16]–[17], two genetic models of dyslipidemia-driven atherosclerosis, as well as in rodents strains characterised by insulin resistance and liver steatosis [16]–[20]. Further on FXR is expressed by cells of innate immunity and both natural and synthetic FXR ligands counter-regulate macrophage activation induced by Toll-like receptor (TLR)-4 ligands [17]. This regulatory effects of FXR on macrophage effector's function is inhibitory in nature and translates in vivo in an attenuated formation of aortic plaques as well as reduced expression of CD36 on circulating monocytes [17].

Because development of therapeutic interventions to effectively counteract the complications of HIV PIs based therapy is especially urgent, we have investigated whether FXR agonism protects against dyslipidemia and vascular injury induced by ritonavir, a widely used HIV-PIs, in rodent models of dyslipidemia-driven atherosclerosis.

## Results

### FXR Agonism Protects Against Dyslipidemia Induced By Ritonavir

HIV-PIs are thought to influence the development of cardiovascular disease primarily through increasing plasma triacylglycerols and cholesterol levels [5]–[8]. Because FXR gene ablations promotes a pro-atherogenic lipid profile and FXR ligands rescue from dyslipidemia in rodents, we have investigated whether FXR agonism attenuates dyslipidemia caused by ritonavir a widely used HIV-PI in mice. The natural FXR ligand CDCA was used because it effectively reduces plasma triacylglycerols levels in dyslipidemic patients [21], [22].

**Figure 1:**
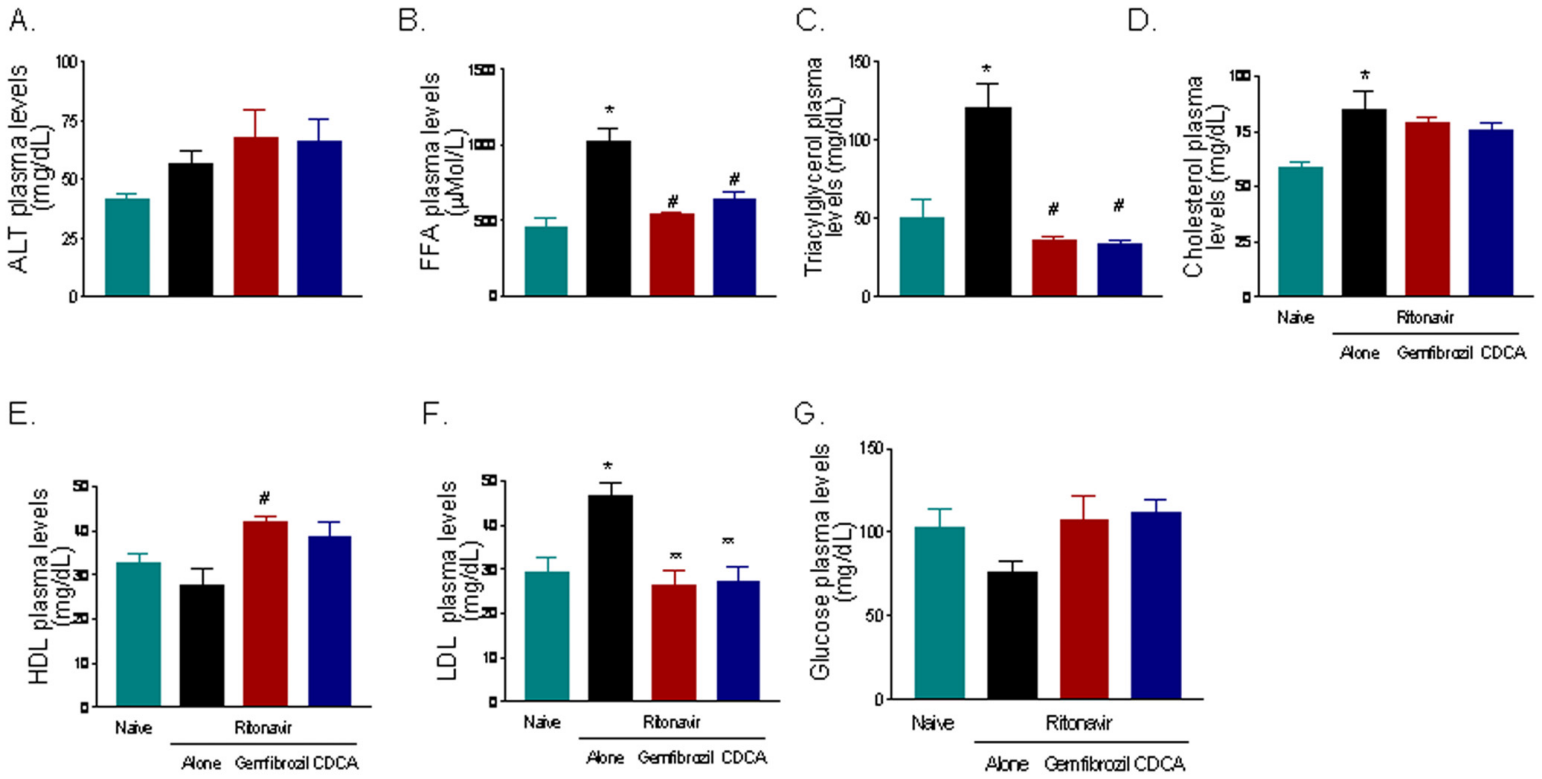
FXR and PPAR α ligands attenuate dyslipidemia induced by the HIV protease inhibitor ritonavir in wild type mice. C57Bl/6 mice at the age of 8 weeks were administered 5 m/kg/day ritonavir alone or in combination with the FXR agonist CDCA, 15 mg/kg/day, or the PPARα agonist gemfibrozil, 100 mg/kg/day, for 10 days. Data shown are plasmatic level of ALT, FFA, triacylglycerols, cholesterol, HDL, LDL and glucose measured at the end of the study period. In each panel, data are mean± SE of 12 animal per group. *P<0.05 ritonavir versus naive; **P<0.05 treatments versus ritonavir alone.

**Figure 2:**
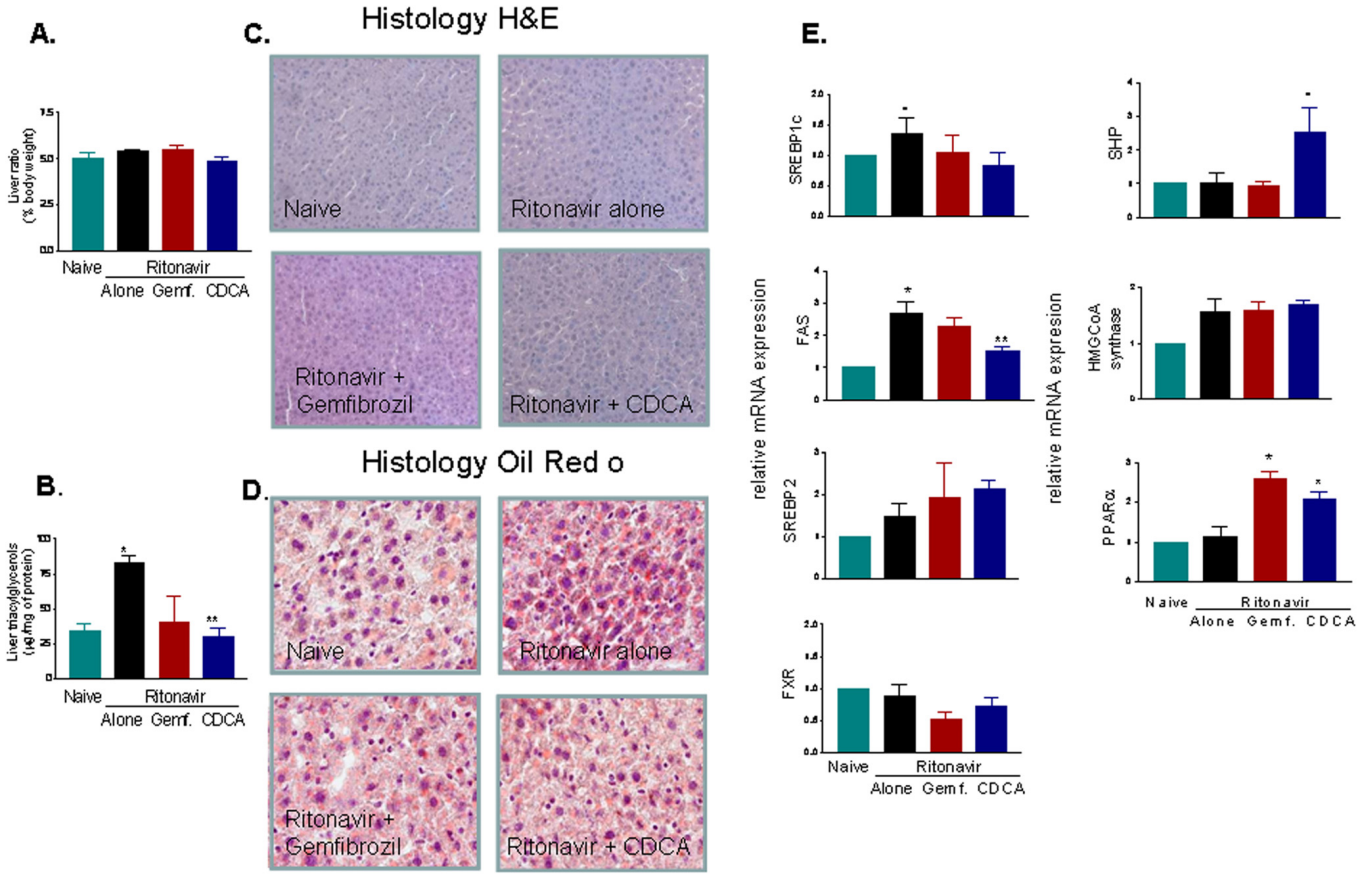
FXR and PPAR α ligands attenuate liver steatosis induced by the HIV protease inhibitor ritonavir in wild type mice. **Panel A.** Liver weight/liver bodyweight ratio after 10 days of administration of ritonavir alone or combination with CDCA or gemfibrozil. **Panel B.** Liver triacylglycerols (µg/mg protein) content. Treating mice with CDCA and gemfibrozil protected against increased liver triacylglycerols accumulation caused by ritonavir. In both panel A and B, data are mean± SE of 12 animal per group. *P<0.05 ritonavir versus naive; **P<0.05 treatments versus ritonavir alone. **Panel C and D.** Histophatologic analysis of livers obtained from wild type mice administered with ritonavir alone or in combination with CDCA or gemfibrozil. Panel C, H&E staining, and panel D, Oil-Red-O staining, showing liver accumulation of triacylglycerols and moderate steatosis. Treatment of mice with ritonavir (5 mg/kg) caused the appearance of microvescicular steatosis and hepatocyte ballooning with minor portal and lobular inflammation. Administering mice with an FXR and PPARα agonist in combination with ritonavir effectively reduced the severity of the liver microsteatosis. E&O, magnification 20×; Oil red O, magnification 100×. **Panel E.** RT-PCR analysis of liver expression of genes involved in lipid and cholesterol homeostasis and nuclear receptors. Treatment with ritonavir induced the expression of genes involved in triacylglycerols synthesis (SREBP1c and FAS), as well as genes involved in cholesterol synthesis (HMCoA synthase). CDCA effectively reduced SREBP1c and FAS accumulation caused by ritonavir while no changes are were observed in the expression of SREBP2 and HMCoA synthase. Administering mice with CDCA also increased the expression of SHP mRNA. Finally, both CDCA and gemfibrozil increased the liver expression of PPARα mRNA. In each panel, data are mean± SE of 6 animal per group. *P<0.05 ritonavir versus naive; **P<0.05 treatments versus ritonavir alone.

We found that 10 days administration of ritonavir to wild type mice increased plasma triacylglycerols, FFA, cholesterol and LDL. This effect was abrogated by co-treating wild type mice with CDCA, and gemfibrozil, a clinically used PPARα, agonist (Figure 1; p<0.05; n = 5–6 per group). Administering mice with ritonavir had no effect on liver weight and liver weight/liver bodyweight ratio (Figure 2A, n = 5,6; P>0.05), but increased significantly the liver content of triacylglycerols (Figure 2B, n = 5,6; P<0.05). Liver accumulation of triacylglycerols translated in the appearance of liver steatosis as demonstrated by the histopathology analysis shown in Figure 2C. Indeed, staining of liver sections with H&E demonstrates that exposure to ritonavir (5 mg/kg) resulted in a microvescicular steatosis and hepatocyte ballooning with minor portal and lobular inflammation. Administering mice with an FXR and a PPARα agonist in combination with ritonavir effectively reduced the severity of the liver microsteatosis, (Figure 2 C). The morphometric analysis of liver sections stained with Oil red-o, a measure of liver triacylglycerols content, confirmed this pattern and corroborated biochemical tissue analyses (Figure 2D). To gain mechanistic insights on the effect exerted by ritonavir we have profiled the liver expression of genes involved in lipid and cholesterol homeostasis and found that treating mice with ritonavir caused a shift in the liver expression of genes involved in lipid homeostasis including SREBP1c and FAS (Figure 2E; n = 6; P<0.05), and genes involved in cholesterol synthesis, such as SREBP2 and HMCoA synthase, although the later was not statistically significant. Co-treating mice with gemfibrozil and CDCA, attenuated this in pattern. Specifically, CDCA effectively prevented the induction of SREBP1c and FAS caused by ritonavir (Figure 2E, n = 6; p<0.05), thought that no changes were observed in the expression of SREBP2 and HMCoA synthase (Figure 2E). Administering mice with CDCA also increased the expression of SHP mRNA, an nuclear receptor and FXR target gene. Finally, both CDCA and gemfibrozil increased the liver expression of PPARα mRNA (Figure 2E; n = 6; p<0.05 versus ritonavir alone).

### FXR gene Ablation Exacerbates the Dyslipidemia Caused by Ritonavir

**Figure 3:**
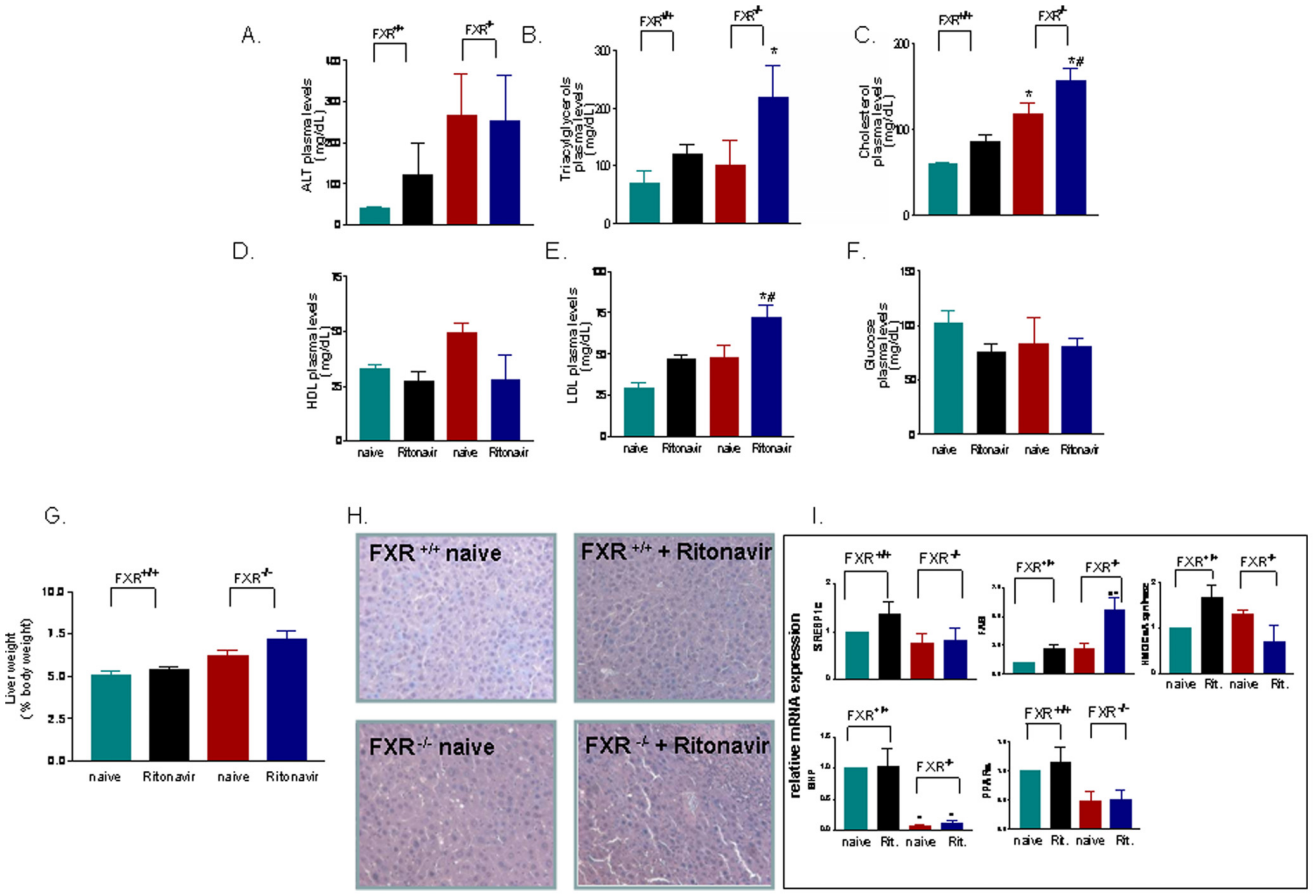
FXR gene ablation worsens the severity of dyslipidemia caused by ritonavir. **Panel A–F.** Plasmatic level of ALT, triacylglycerols, cholesterol, HDL, LDL and glucose in wild type and FXR^−/−^ mice administered ritonavir, 5 mg/kg/day, for 10 days. In each panel, data are mean± SE of 6 animal per group. *P<0.05 versus wild type naive; **P<0.05 treatments versus ritonavir wild type. **Panel G.** Liver weight/liver body weight ratio after 10 days administration of ritonavir. In each panel, data are mean± SE of 8 animal per group. **Panel H.** Liver histophatology. H&E staining of liver sections obtained from FXR^+/+^ and in FXR^−/−^ mice. Magnification 20×. **Figure I.** RT-PCR analysis of liver expression of genes involved in lipid and cholesterol homeostasis and nuclear receptors. Treatment with ritonavir caused a significant dysregulation of FAS mRNA expression in FXR^+/+^ and FXR^−/−^ mice. FXR^−/−^ mice show a severe reduction of liver expression of SHP and PPARα mRNA that is insensitive to ritonavir. In each panel, data are mean± SE of 6 animal per group. *P<0.05 versus wild type naive; **P<0.05 treatments versus ritonavir wild type.

Because these data indicate that FXR agonism effectively reduced the severity of dyslipidemia caused by ritonavir, we have investigated in an independent set of experiments whether FXR gene ablation would exacerbate the dyslipidemia caused by the administration of this HIV-PI. As shown in Figure 3, FXR gene ablation exacerbated the severity of dyslipidemia caused by ritonavir (Figure 3A–F, n = 6–8; P<0.05). In addition, mice harbouring a disrupted FXR gene had a more pronounced liver steatosis (Figure 3H) and developed a specific pattern of liver expression of genes involved in lipid and cholesterol metabolism. The main feature of the later being the robust dysregulation of FAS mRNA expression and the marked reduction of the expression of PPARα and SHP mRNAs (Figure 3I; n = 6; p<0.05).

### FXR Activation Protects Against Atherosclerosis Development In ApoE^−/−^ Mice Treated With Ritonavir

**Figure 4:**
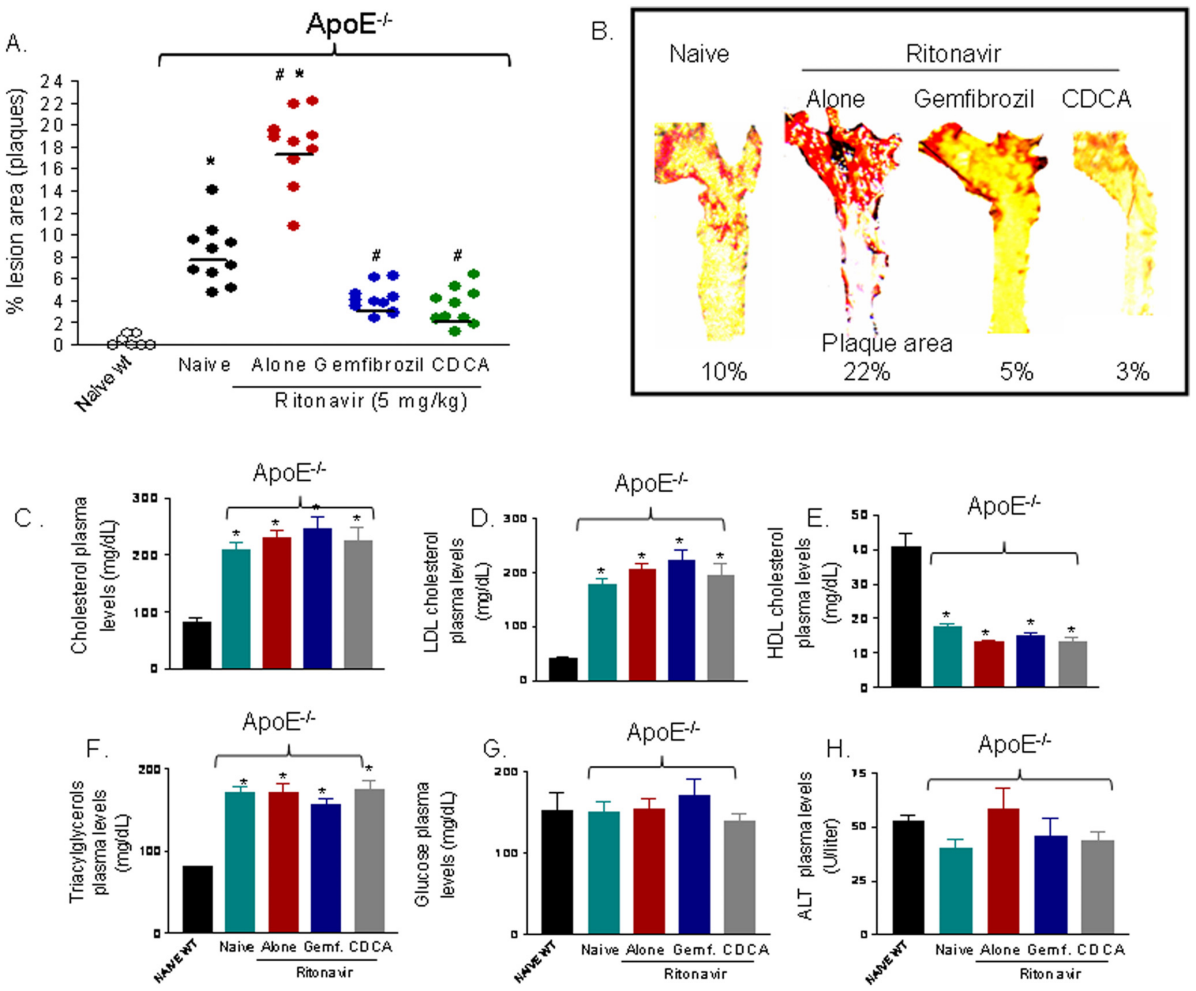
FXR and PPAR α ligands protects against aortic plaques formation induced by the HIV-PI ritonavir to ApoE^−/−^ mice. **Panel A.** Administration of ritonavir (5 mg/kg/day) to ApoE^−/−^ for 12 weeks increases the size of aortic plaques expressed of ratio of plaque surface area to vessel surface area. The effect was significantly attenuated by co-treating mice with gemfibrozil or CDCA. Individual data of 10 animals per group are shown. *P<0.05 versus wild type naive; #P<0.05 treatments versus ritonavir. **Panel B.** Pseudo-colour representation of aortic plaques from individual animals. The images show the plaque surface from individual animals, each one representative of a specific experimental group. The lipids in the vessel wall were staining with Sudan IV, as described in Material and Methods, and shown in red. **Panel C–H.** Plasmatic levels of ALT, triacylglycerols, cholesterol, HDL, LDL and glucose in ApoE^−/−^ mice. ApoE^−/−^ mice show increased plasma cholesterol, triacylglycerols and LDL plasma levels and decreased HDL plasma levels in comparison to their wild-type counterparts (n = 10; * P<0.01 versus naive wild type). Twelve-weeks administration of ritonavir failed to change this pattern and pharmacological treatments were unable to attenuate the pro-atherogenetic lipid profile observed in ApoE^−/−^ administered ritonavir (n = 10).

Treating ApoE^−/−^ mice with the HIV-PI ritonavir alone or in combination with CDCA, and gemfibrozil for three months had no effect on mice body weight and mortality (data not shown). Administration of ritonavir (5 mg/kg/day) to ApoE^−/−^ however, resulted in a dramatic increase in the extension of aortic plaques (Figure 4A, n = 10 per group; P<0.05). The development of aortic plaques was significantly attenuated by co-treating ApoE^−/−^ mice fed ritonavir with CDCA and gemfibrozil (P<0.05 and P<0.01 respectively; Figure 4A). Analysis of Sudan IV staining of whole aortas, to measure neutral lipid content in the vessel, demonstrated that ritonavir exacerbated plaque lipid accumulation in ApoE^−/−^ mice and that this effect was robustly attenuated by both CDCA and gemfibrozil (Figure 4B; P<0.01).

When compared to their wild-type counterparts, ApoE^−/−^ mice showed a twofold increase in plasma cholesterol and triglycerides levels, a fourfold increase in plasma LDL levels and threefold decrease in plasma HDL levels (Figure 4C–H; n = 10; P<0.01). Twelve-week administration of ritonavir failed to modify this pattern and pharmacological treatments were also unable to attenuate this pro-atherogenic lipid profile (Figure 4; n = 10; P<0.05 versus ritonavir). Because, the later finding was in a striking contrast with previous observations demonstrating that FXR agonism protects against development of pro-atherogenic lipid profile in ApoE^−/−^, we have investigated whether resistance to the ipolipemic effects of CDCA and gemfibrozil could be ascribed to the administration of ritonavir. For this purpose ApoE^−/−^ mice were treated with CDCA and gemfibrozil for 12 weeks without ritonavir. Results of these experiments demonstrated that both CDCA and gemfibrozil were effective in reducing plasma levels of triacylglycerols and total cholesterol in ApoE^−/−^ in the absence of ritonavir (Figure S1), strongly supporting the view that the HIV-PI was responsible for the inability of FXR and PPARα agonists to rescue ApoE^−/−^ mice from development dyslipidemia. Finally, ritonavir treatment had no effect on plasma glucose (Figure 4G).

**Figure 5:**
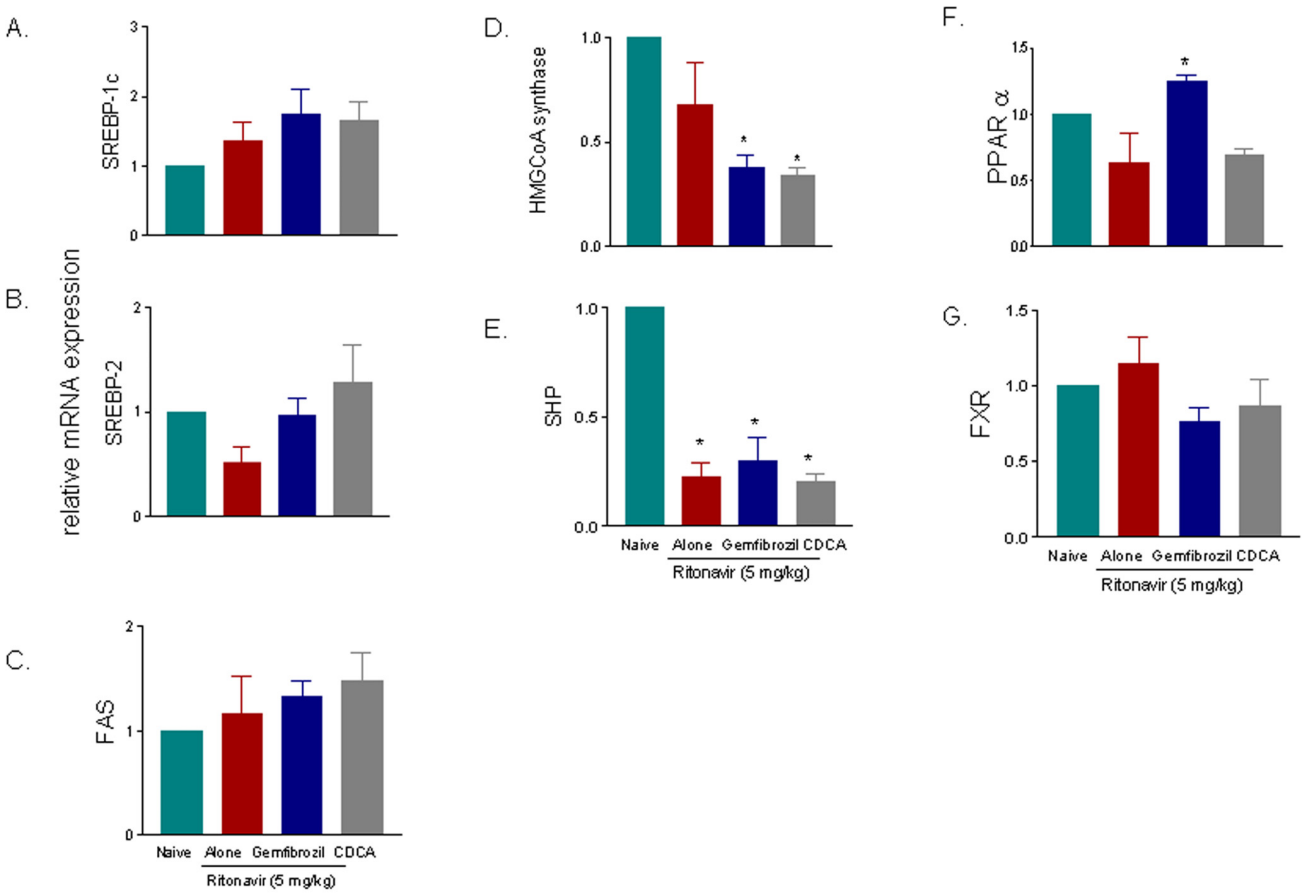
FXR and PPARα ligands does not impact on liver expression of genes involved in lipid and cholesterol metabolism in ApoE^−/−^ mice administered the HIV-PI ritonavir. In each panel, data are mean± SE of 6 animal per group. *P<0.05 versus wild type naïve mice.

Treating ApoE^−/−^ mice with ritonavir caused no changes in the pattern of liver expression of genes involved in lipid and cholesterol metabolism, and similarly, no changes in the expression of these gene was detected in mice administered CDCA and gemfibrozil (Figure 5A–G).

### FXR Agonism Counter-regulates the effect of Ritonavir on CD36 expression *In vivo* and *in vitro*

**Figure 6:**
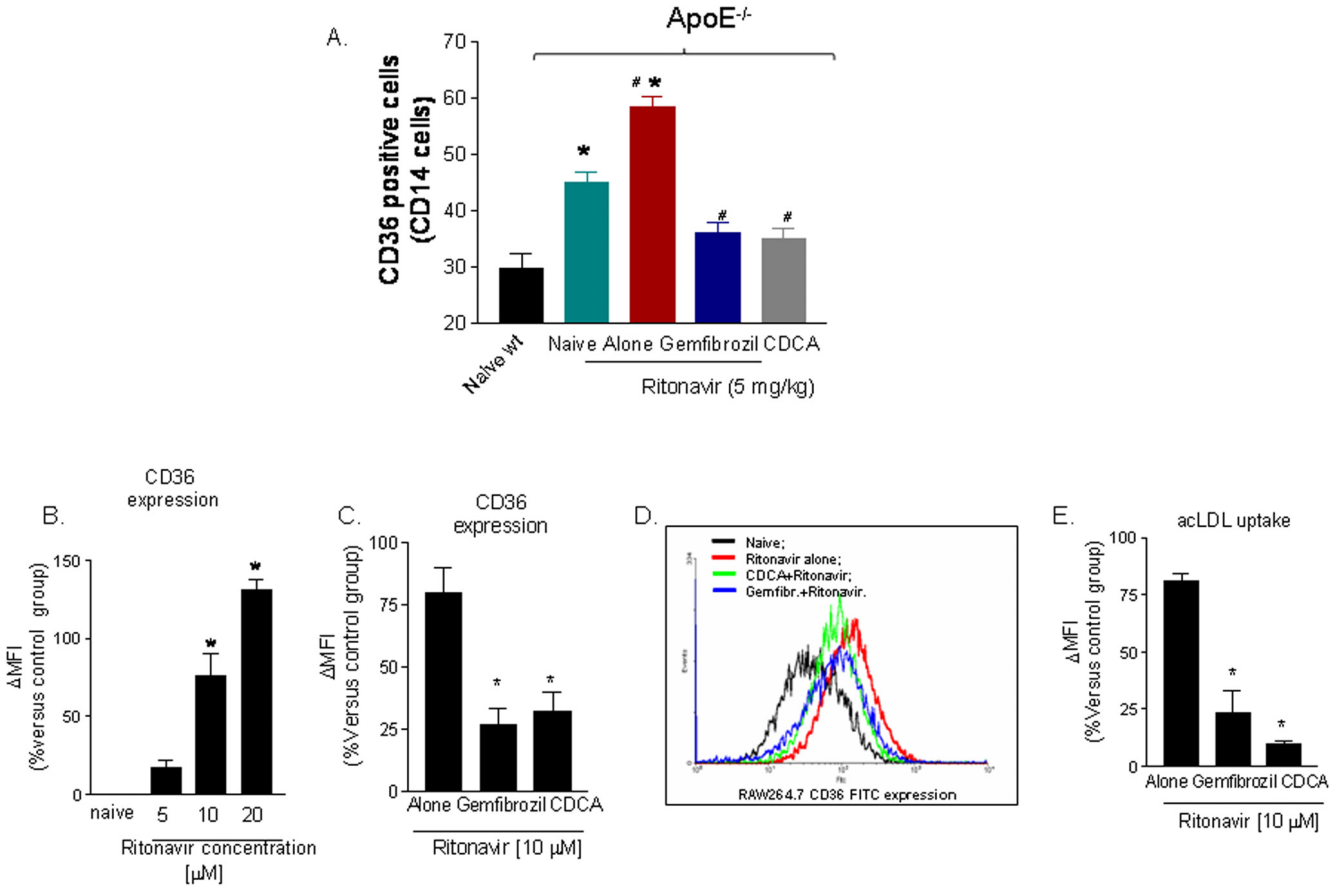
FXR and PPAR α ligands revert the dsysregulation of CD36 expression caused by administration of the HIV-PI ritonavir to ApoE^−/−^ mice. **Panel A.** CD36 expression on circulating was induced by administering ApoE−/− mice with ritonavir 5 mg/kg/day for 12 week (n = 6 P<0.05) s. Co-treatment with gemfibrozil or CDCA abrobated this effect. Data are mean± SE of 12 animal per group. *P<0.05 versus wild type naive; **P<0.05 versus ApoE^−/−^; # P<0.05 treatments versus ritonavir alone. **Panel B–E. Ritonavir, gemfibrozil and CDCA exert opposite effects on CD36 expression/function in cultured macrophages (RAW264.7 cells).** Ritonavir treatment increased the cell surface expression of CD36 on cultured macrophages in a concentration-dependent manner (Figure 6B, n = 5; P<0.05) and co-treatment of the cells with gemfibrozil and CDCA (Figure 6 C,D n = 5; p<0.05) reduced CD36 expression caused by 10 µM ritonavir by approximately 75% (Figure C,D; n = 5; P<0.05). Exposure of RAW264.7 cells to 10 µM ritonavir increased CD36-mediated uptake of acetylated LDL (ac-LDL). This effect was abrogated by gemfibrozil, 250 µM, and CDCA, 50 µM (n = 5; P<0.05 versus ritonavir alone).

As shown in Figure 6, blood monocytes isolated from ApoE^−/−^ had a significant higher expression of CD36 in comparison to wild type naive mice (44.9±1.9 versus 29.6±2.8; n = 6 P<0.05). Treating ApoE^−/−^ mice with ritonavir for 12 weeks caused a further increase of CD36 expression on circulating monocytes (from 44.9±1.9 to 58.3±3.3; n = 6; P<0.05). However, co-treating ApoE^−/−^ mice with CDCA and gemfibrozil resulted in a robust attenuation of CD36 expression on monocyte's cell surface and completely reversed the effect exerted by ritonavir (Figure 6A; P<0.05 and P<0.01 versus ApoE^−/−^ naïve and ApoE^−/−^ plus ritonavir, respectively).

Previous studies have shown that HIV-PIs disrupt cellular lipid homeostasis by increasing the levels of SREBPs and by inducing a SREBP-related increase in CD36 expression and lipid uptake [9], [10], [11], [23]. We have therefore examined whether HIV-PIs directly modulate CD36 expression in cultured macrophages. For this purpose RAW264.7 cells, a macrophage cell line, were directly challenged with HIV-PIs. Results from this experiment (Figure 6B) demonstrate that ritonavir (Figure 6B–D) and atazanavir, another clinically used HIV-PI (Figure S2), increased the cell surface expression of CD36 in a concentration-dependent manner (Figure 6B, n = 5; P<0.05), and that this effect was abrogated by co-treating the RAW264.7 cells with CDCA and gemfibrozil (Figure 6C, n = 5; p<0.05). Modulation of CD36 expression by FXR and PPARα agonism correlated with a reduced uptake ac-LDL by cultured macrophages. Thus, CDCA and gemfibrozil effectively attenuated ac-HDL uptake caused by ritonavir (Figure 6D, n = 5; P<0.05 versus ritonavir alone). In addition, exposure of RAW264.7 cells to GW4064, a non steroidal FXR ligand, attenuated CD36 induction caused by ritonavir and atazanavir (P<0.05; n = 4; Figure S2).

**Figure 7:**
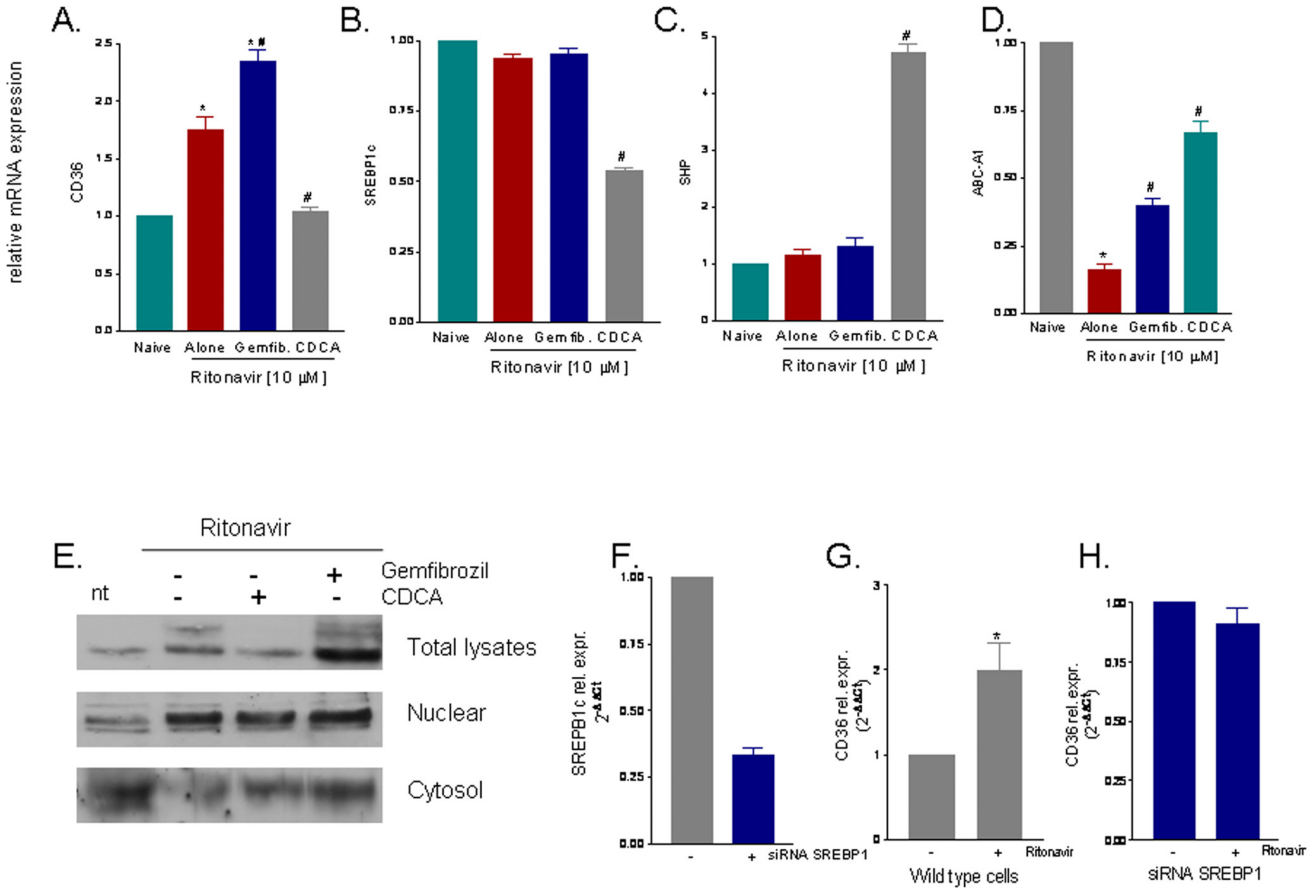
Distinct patterns of gene and protein expression are induced by FXR and PPARα ligands in macrophages challenged with ritonavir. **Panel A–D.** RT-PCR analysis of gene expression induced by exposure RAW264.7 cells to ritonavir alone or in combination with a PPARα or FXR ligand. Ritonavir slightly increased expression of CD36 mRNA (Figure 7A) while CDCA, but not gemfibrozil, completely abrogated this effect. In addition exposure CDCA, but not to gemfibrozil, reduced SREBP1c mRNA and increased the expression of SHP mRNA (P<0.05 versus ritonavir). In each panel, data are mean± SE of 5 separate experiments. **Panel E.** Detection of nuclear translocation of SREBP1c by Western blot analysis. Treatment of RAW264.7 cells with ritonavir increased protein level of SREBP1c and caused its nuclear translocation. Activation of FXR with CDCA reduced total SREBP1c protein and prevented its nuclear translocation. Data shown is representative of three other experiments showing the same pattern. **Panel F–H.** Transfection of RAW264.7 macrophages with an anti-SREBP1c siRNAs resulted in a robust downregulation of SREBP1c mRNA (≈70%, P<0.05 versus naive cells). The knock-down of SREBP1c abrogated the ability of ritonavir to induce CD36 (panel 7H versus panel G). In each panel, data are mean± SE of 4 separate experiments.

We have then examined the mechanism involved in CD36 induction caused by ritonavir. As illustrated in Figure 7A exposure of RAW264.7 to ritonavir resulted in robust increase in the expression of CD36 mRNAs (P<0.05, n = 4). This pattern was significantly shifted by treating the cells with CDCA. Thus exposure of RAW 264.7 cells to the FXR ligand, but not to gemfibrozil, attenuated induction of CD36 caused by ritonavir and reduced the expression of SREBP1c mRNA by approximately 50%, while caused a 4–5 fold increase in SHP mRNA (Figure 7 B–C; P<0.05, versus ritonavir). Importantly, both gemfibrozil and CDCA increased the expression of ABCA1, a gene involved in cholesterol secretion by macrophages (Figure 7D; P<0.05 versus ritonavir alone).

In addition to these genomic effects, ritonavir interfered with cell handling of SREBP1c. Thus exposure of RAW264.7 to ritonavir leads to its translocation to the nucleus, an event that is associated with binding of this regulatory factor to its target genes (Figure 7E), This effect was robustly attenuated by CDCA but not by gemfibrozil, indicating that FAXR and PPARα agonists act on different steps of post-translation regulation of CD36.

The expression of the CD36 gene should be properly regulated to ensure its functions [24], [25]. Because an involvement of SREBPs in the activation of CD36 is an interesting possibility of coordination of a homeostatic balance between fatty acids and sterols, we have examined whether SREBP1c mediates CD36 mRNA upregulation caused by HIV-PIs. For this purpose expression of SREBP1c in RAW264.7 macrophages was knocked down by transfecting the cells with a validated anti-SREBP1c siRNAs. As shown in Figure 7F, transfecting the cells with anti-SREBP1c siRNAs resulted in robust downregulation of SREBP1c mRNA (approximately 70%, P<0.05 versus naive cells). The knockout of SREBP1c abrogated the ability of ritonavir to induce CD36 (Figure 7H versus 7G) highlighting a putative role of SREBP1c in mediating the effect of ritonavir on CD36. To gain further insights on the mechanisms involved in the regulation of CD36 by SREBP1c, we have carried out an analysis of the CD36 promoter in the search for a putative binding site for SREBP1c. Because this analysis revealed the presence of a putative sterol regulatory element (SRE)-binding consensus in the promoter of CD36, we have performed a ChIP assay and PCR amplification using primer pairs that cover the putative CD36-SRE consensus site located at −76 in the CD36 promoter. Results from these experiments, however, were negative, indicating that in RAW264.7 cells induction of CD36 expression caused by ritonavir is not mediated by a direct binding of SREBP1c to the CD36 promoter and is likely mediated by activation of additional mechanisms (Figure S3).

## Discussion

FXR is a nuclear receptor that functions as a bile acid sensor and plays a regulatory role in bile acids, cholesterol and lipid homeostasis [26]. Previous studies have shown that FXR might be an interesting target for preventing development of atherosclerosis. This contention results from the aggregate evaluation of the fact that FXR gene ablation results in a pro-atherogenic lipoproteins profile [15] and FXR and ApoE double knockout male mice fed an high fat diet develop an accelerated atherosclerosis in comparison to ApoE^−/−^ single knock out mice [27]. In addition, we and others have demonstrated that FXR activation protects against atherosclerosis plaque formation in ApoE^−/−^ and LDLr^−/−^ mice [16], [17].

**Figure 8:**
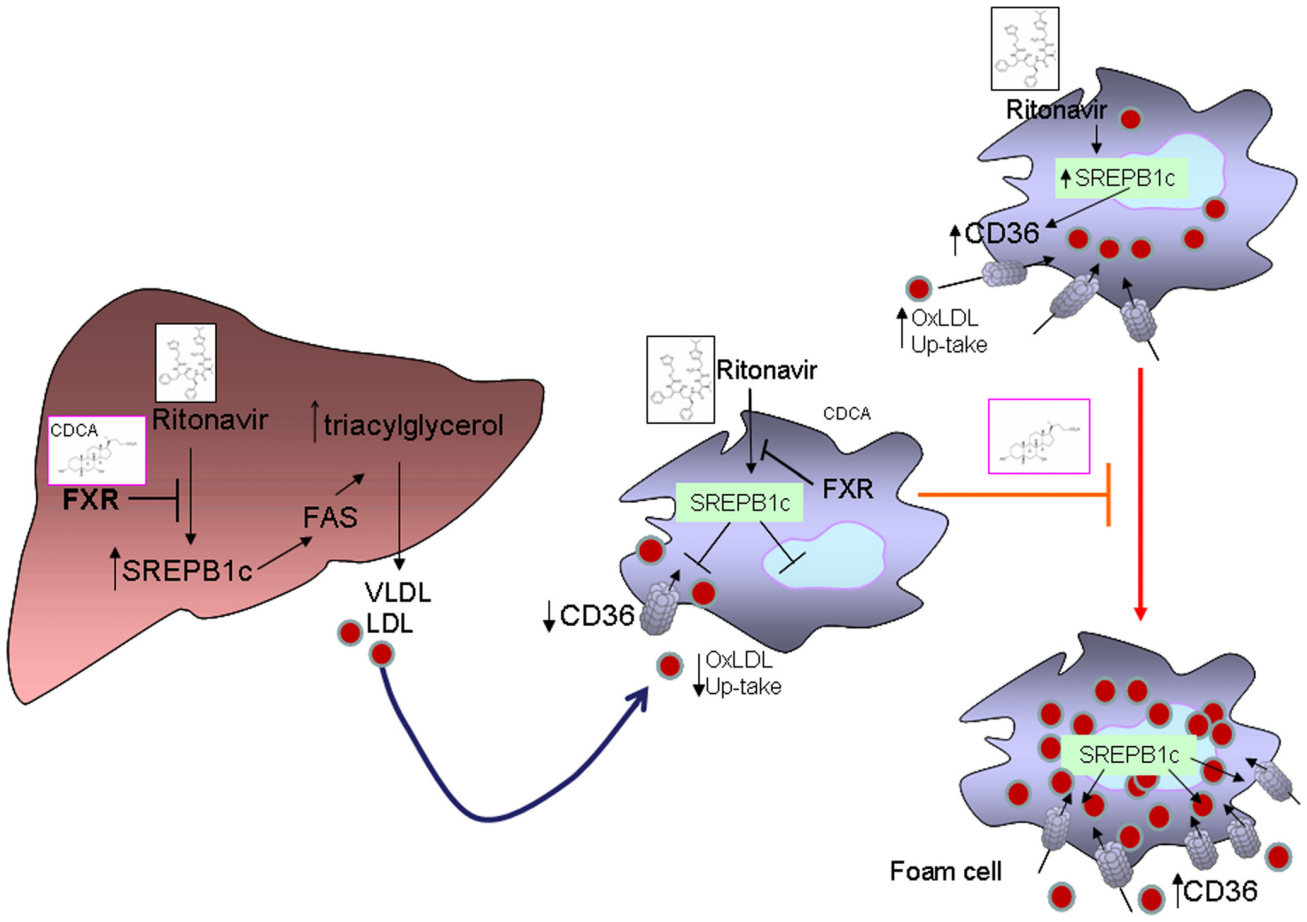
Diagramatic representation of the effect exerted by FXR and PPARα ligands on dyslipidemia and accelerated atherosclerosis caused by the HIV-PI ritonavir. Exposure to ritonavir causes SREBP1c activation in the liver and monocytes. In circulating monocytes ritonavir causes a SREBP1c-dependent induction of CD36. Increased expression of CD36 leads to uptake of ac-LDL and favours the acquisition of an activated phenotype by monocytes and their transformation into foam cells. Activation of FXR, reduces CD36 expression on macrophages by interfering with SREBP1c. Knocking down SREBP1c by siRNAs abrogates the activation of CD36 caused by ritonavir. Activatory pathways are shown in black and inhibitory pathways in red.

In the present study we have provided evidence that FXR activation with CDCA effectively protects against dyslipidemia and atherosclerosis development in rodents fed ritonavir, a widely used HIV PI (Figure 8). Despite the fact that synthetic and semi-synthetic FXR ligands are currently investigated [26], we have decided to use CDCA, the natural FXR ligand, for the following reasons. The prevalence of HIV infection is growing in undeveloped countries [1]–[3] and the cost of co-medications to prevent HAART's related side effects add significantly to the already elevated cost for HAART itself. Our aim was therefore to exploit a drug that would have beneficial effects without increasing further the costs of HIV management. CDCA has a low cost and is the only FXR ligand available worldwide that has been demonstrated effective in reducing dyslipidemia in clinical settings [21], [22].

### FXR activation protects against development of dyslipidemia in wild type mice

HAART has had a dramatic effect in reducing morbidity and mortality associated with HIV-1 infection. However, concern has been raised in relation to the increased risk of coronary heart disease linked to chronic use of HAART. Findings from the Data Collection on Adverse Events of Anti-HIV Drugs (DAD) study indicate that the incidence of myocardial infarction in HIV infected persons increases with long-term exposure to HAART [2]–[4], [8]. In a large prospective observational study including 23,437 patients the incidence of myocardial infarction increased from 1.53 per 1000 person-years in those not exposed to HIV PIs to 6.01 per 1000 person-years in those exposed to HIV PIs for more than 6 years. After adjustment for exposure to the other drug class and established cardiovascular risk factors (excluding lipid levels), the relative rate of myocardial infarction per year of HIV PIs exposure was 1.16 (95% confidence interval [CI], 1.10 to 1.23). Despite adjustment for serum lipid levels further reduced the effect of exposure to each drug class to 1.10 (95% CI, 1.04 to 1.18), these results demonstrate that exposure to protease inhibitors is associated with an increased risk of myocardial infarction which is only partly explained by dyslipidemia. In contrast, there is no evidence that the use of non-nucleoside reverse-transcriptase inhibitors included in the HAART increases the risk of myocardial infarction [28], [29].

Beside the DAD study, smaller studies have provided evidence that long-term use of HAART regimens based on HIV PI lopinavir/ritonavir increases the levels of triglycerides and results in a pro-atherosclerotic lipoprotein profile [5]–[7]. These studies indicate that the hyperlipidemia produced by HIV PI is primarily attributable to an excessive FFA mobilization occurring because of insulin resistance in the adipose tissue, resulting in increased VLDL–triglyceride production and apoB synthesis. Likewise, t in fed mice, ritonavir had no effect on serum glucose and cholesterol, whereas triglyceride and fatty acids increased by 57% to 108% [30]. In fasted mice, ritonavir increased serum glucose by 29%, cholesterol by 40%, and triglyceride by 99%. The hyperlipidemia was attributed to elevated FFA and cholesterol synthesis by the liver and adipose tissue caused by activation of SREBP1c and 2, whereas studies in cultured liver cells support the notion that ritonavir might cause a decreased degradation of nascent apoB attributable to inhibition of the 20S proteasome.

In the present study we have confirmed that administration of ritonavir to wild type mice increases triacylglycerols synthesis. A two-fold increase in FFA, triacylglycerols and LDL plasma levels was observed in mice administered 5 mg/kg/day ritonavir for 10 days, indicating that this model reproduces all major metabolic features observed in HIV infected persons administered liponavir/ritonavir. A major observation of the present study was the demonstration that such lipid abnormalities are highly dependent on the activity of the nuclear receptor FXR. Thus, not only activation of FXR with CDCA protects against development of dyslipidemia caused by ritonavir, but feeding ritonavir to FXR^−/−^ mice resulted in an exacerbation of biochemical derangements caused by the HIV PI in wild type mice. Together, these observations provide a strong support to the notion that FXR might be a novel therapeutic target for preventing/treating lipid metabolism abnormalities caused by HIV PI.

Feeding with FXR agonists attenuates dyslipidemia in a variety of rodent models [16]–[20]. A key regulatory pathway involved in these effects is the inhibition of SREBP1c. Administering rodents with natural and synthetic FXR ligands inhibits the expression of SREBP1c in the liver. Originally, this effect was thought to be mediated by SHP, an atypical nuclear receptor that lacks a DNA binding domain. Based on studies in *SHP*-deficient mice, it has been proposed that the induction of SHP mediates the downregulation of SREBP-1c expression in response to bile acids treatment [18], [19], [31]. Counter-intuitively, however, a decreased, and not increased, SREBP-1c mRNA is observed in *ob/ob/SHP* ^−/−^ double mutants [32]. In addition to the observation that transgenic mice constitutively expressing SHP in the liver exhibited an enhanced expression of SREBP-1c mRNA levels and a concomitant accumulation of hepatic triglycerides [33], these data suggest that also a SHP-independent mechanism is activated by FXR ligands. In the present study we have shown that CDCA induced the liver expression of SHP and was as effective as the PPARα ligand gemfibrozil in correcting lipid abnormalities caused by ritonavir. The two agents exerted a comparable FFA-, triacylglycerols- and LDL-lowering effect. Both agents had minor effect on cholesterol plasma levels, confirming that ritonavir increases LDL levels primarily by inducing FFA and triacylglycerols synthesis.

A further confirmation of this view comes from results of experiments carried out in FXR^−/−^ mice. Indeed, challenging FXR^−/−^ mice with ritonavir exacerbated the severity of dyslipidemia induced by ritonavir causing a two-fold increase in FFA, triacylglycerols and LDL plasma levels in comparison to wild type mice administered the HIP-PI. Administering FXR^−/−^ mice with ritonavir also increased cholesterol and reduced HDL levels. The latter effect is commonly observed in response to FXR activation, and it is still unclear whether it holds any potential for detrimental side effects in humans.

Analysis of the expression of regulatory genes in the liver revealed that ritonavir had a minor effects on expression of SREBP1. However, expression of this gene was reduced in mice administered CDCA. Our results are consistent with previous observations indicating that ritonavir increases SREBP1c by reducing its degradation and by causing its stabilization at the promoter of target genes [9], [10]. In summary these results suggest that activation of FXR reverts lipid abnormalities caused by ritonavir and that FXR gene ablation exacerbates the dyslipidemia caused by this HIV PI.

### FXR activation protects against atherosclerosis development in ApoE^−/−^ mice fed ritonavir

An important observation we made is that, in contrast to wild type mice, 12-weeks administration of ritonavir (5 mg/kg/day) to ApoE^−/−^ mice failed to exacerbate the dyslipidemic phenotype of these mice and that, while pharmacological treatments were effective in protecting against accelerated atherosclerosis, both the FXR and PPARα ligand had no effect on serum lipoprotein profile. The lack of the effect on lipid metabolism was confirmed by the analysis of the expression of regulatory genes in the liver. Thus, in striking contrast to what observed in wild type mice, both CDCA and gemfibrozil failed to modulate the liver expression of SREBP1c, SREBP2and FAS. In addition, both treatments had no effects on liver expression of PPARα and FXR. Thought that CDCA increased the liver expression of SHP.

In a previous study we have demonstrated that activation of FXR, by a semi-synthetic derivative of CDCA and PPARγ reduces the liver expression of SREBP1c and its target genes including FAS in ApoE^−/−^ mice, administered a non-fat diet [17]. In the present study we have confirmed that CDCA similarly to its derivative 6-ethyl CDCA, protects against development of a dyslipidemic phenotype in ApoE^−/−^ mice not administered ritonavir (Figure S1), strongly indicating that feeding ApoE^−/−^ mice with an HIV PI introduces a further distortion of the metabolic regulation that can not be reverted by FXR and PPARα ligand in this model.

The fact that CDCA protects against atherosclerotic plaque formation in ApoE^−/−^ despite it had no effect on lipid profile, indicates that FXR activation might impact on expression on pro-inflammatory factors [34]–[40]. Thus, *in vivo* and *in vitro* studies, have demonstrated that HIV PIs increase atherosclerotic lesions by a direct action on macrophages [12]. Macrophages play an essential role in atherosclerosis [41]. Gene-deletion and bone marrow–transplantation experiments have provided evidence that macrophages scavenger receptor A and CD36 exert non dispensable effects in mediating the uptake of oxidized LDL by macrophages and promoting the development of atherosclerosis [42], [43]. CD36 is the primary mediator of cholesterol accumulation in atherosclerosis induced by HIV PIs [12]. One important observation we made is that while administration of ritonavir to ApoE^−/−^ mice increased the percentage of CD36 positive monocytes, chronic administration of CDCA caused a robust reduction in the percentage of CD14/CD36 positive cells in ApoE^−/−^ mice treated with ritonavir. A similar pattern was observed when CD36 mRNA expression was measured in the aortic plaques. Similarly to CDCA, gemfibrozil was also effective in attenuating CD36 expression in the aorta and circulating macrophages. These data are consistent with our previous observations indicating that activation of FXR attenuates the expression/function of CD36 in ApoE^−/−^ naive mice.

The above results were confirmed by in vitro studies. Thus, while treating RAW264.7 macrophages with ritonavir and atazanivar resulted in a concentration-dependent induction of CD36 expression, gene and protein, this effect was completely abrogated by treating the cells with CDCA and GW4064 (a synthetic FXR agonist). The concentration of ritonavir used in this experiment is clinically relevant. Indeed, while plasma concentrations of ritonavir in HIV infected patients treated with ritonavir 100 mg/day twice a day are ≈1 µM, ritonavir accumulates in the peripheral blood mononuclear cells reaching concentrations of ≈20 µM. Importantly, while CDCA and GW4064 had no effect on basal expression of CD36 both agents were effective in reducing CD36 protein expression induced by 10 µM of ritonavir as measured by flow cytometry. Further on, modulation of CD36 expression was functionally correlated with a reduction of ac-LDL uptake by macrophages.

Several of our results indicate that FXR directly antagonizes the effect of ritonavir on CD36 by interacting with SREBP1c. Exposure to ritonavir induces the expression of SREBP1c mRNA as well as its nuclear accumulation. Our results are therefore a variation of previous studies reporting that ritonavir inhibits SREBP1c degradation [9]. The genomic effects of ritonavir could be explained by several mechanisms. In macrophages, HIV PIs activate the unfolded protein response (UPR) which is involved in the regulation of CD36 expression an event that has been involved in foam cell formation [11]. Despite FXR ligands have no effect on post-translational handling of SREBP1c we have found that FXR ligation reduces total SREBP-1c, protein and mRNA levels, by approximately 50% therefore reducing its nuclear translocation. In the present study we have found that CDCA causes a 4–5 fold increase of SHP mRNA and we have documented an inverse correlation between SHP and SREBP-1 expression in RAW264.7 cells exposed to CDCA. These data are consistent with the finding that FXR activation by natural and synthetic agonists represses the expression of SREBP-1c and its lipogenic target genes in mouse primary hepatocytes and in liver [18]–[20], [33]. One important observation we made is that exposure of EAW264.7 cells to CDCA resulted in robust induction in the expression of ABAC1, a gene whose protein product mediates cholesterol efflux from macrophages. The ability of FXR to induce ABCA 1 expression could be of mechanistic relevance in explaining the anti-atherogenic effects of CDCA [44].

Several experimental data link SREBP responsive elements (SRE) to the regulation of CD36 gene expression. Thus, knocking down the expression of SREBP1c greatly attenuated the induction of CD36 caused by ritonavir. However, despite we have identified a putative SRE-binding site in the promoter of mouse CD36 gene, we have been unable to show a functional role for this SRE in RAW264.7 cells exposed to ritonavir, indicating that ritonavir induces CD36 expression by multiple, yet not completely identified, pathways.

Despite the present study demonstrates that FXR activation attenuates dyslipidemia caused by ritonavir, this is a preclinical investigation and its translational relevance should be viewed with some cautions. Indeed, the causes of dyslipidemia in HIV infected patients taking a PI are not completely clarified.

In addition to the above described mechanisms, alternative regulatory pathways could have been activated by exposure of macrophages to ritonavir. The pregnane –x-receptor (PXR) is a nuclear receptor and xenobiotic sensor and is a ritonavir target gene. Previous studies have shown that PXR directly activates SREBP1c and CD36 by binding to PXR responsive elements in the CD36 promoter. However, we have ruled out a possible role for PXR in the negative regulatory effects exerted by FXR agonists on this scavenger receptor, because FXR is a known PXR inducer [45], [46]. Thus, exposure to FXR ligand would results in the induction of PXR, that is a positive regulatory gene for CD36.

In conclusion, we have provided evidence that FXR activation exerts anti-atherosclerotic effects in ApoE^−/−^ mice treated with an HIV PI and that these effects are independent on modulation of lipid metabolism, but requires the regulation of CD36 expression and activity. These data suggest that CDCA by modulating SREPBP1c expression specifically antagonizes the effects of ritonavir of CD36, a mechanism involved in dyslipidemia caused by HIV-PI.

## Materials and Methods

### Animal and treatments

The present study involves the use of rodents. All experiments were performed in accordance to international and Italian guidelines for use of laboratory animals. The present protocol was approved by the Italian Minister Health and conforms to national guidelines. The ID for this project is #11/2010-B. The authorization was released to prof. Stefano Fiorucci, as a principal investigator, on January 25, 2010. Mice were maintained in a temperature controlled facility with a 12-hour light/dark cycle and were given free access to food and water. Eight weeks old wild type and FXR^−/−^ mice were randomized into four groups (N = 10): group 1, no treatment; group 2 intraperitoneally administration of ritonavir 5 mg/Kg/day in a total volume of 100 µl alone; groups 3 and 4 received ritonavir (5 mg/Kg/day) in combination with CDCA (20 mg/kg/day by gavage) and gemfibrozil (100 mg/kg/day by gavage) respectively. While FXR null mice, eight weeks old, were randomized into two groups (N = 10): group 1, no treatment and group 2 intraperitoneally administration of 100 µl ritonavir (5 mg/Kg/day) alone. All drugs were administered 14 days. During the experiment mice were weight once a week. At the end of the experiment, 12-hour fasted animals received the latest administration of the specific agent and 6 hours later were anesthetized with sodium pentobarbital and then sacrificed. Blood and livers were collected. Serum content of total cholesterol, triglyceride, HDL, LDL, glucose and alanine aminotransferase were measured by enzymatic assays (Wako Chemicals; Osaka, Japan).

ApoE^−/−^ male mice on a C57BL6/J background and wild type C57BL6/J male mice were from the Harlan Nossan (Udine, Italy). Ten weeks old ApoE^−/−^ mice were randomized into four groups (n = 10 per group): group 1, no treatment; group 2 mice were administered intraperitoneally with 100 µl of a solution containing 5 mg/Kg/day ritonavir; groups 3 and 4 mice were administered with r ritonavir (5 mg/Kg/day) in combination with CDCA (15 mg/kg/day by gavage) or gemfibrozil (100 mg/kg/day by gavage) respectively. All drugs were administered five days a week for 12 weeks. During the experiment mice were weight once a week. At the end of the experiment animals were fasted for 16 hours, anesthetized with sodium pentobarbital before harvesting blood for subsequent lipid measurements and monocytes/macrophages isolated for the measurement of CD36 expression by flow cytometry. Liver samples for RNA isolation were immediately snap frozen. Liver samples for histology were embedded in paraffin. The aortas were processed for enface atherosclerotic lesion coloration. This protocol was repeated three fold and results on one experiment are shown. In the second set of experiments (8 mice per group), animals received the same treatments described above and at the end blood was collected for RNA isolation and monocytes/macrophages isolated for CD36 expression.

### Quantification of atherosclerotic plaques and liver histology

The heart and upper section of the aorta were removed from animals, cleaned of peripheral fat under a dissecting microscope, and sectioned parallel to the atria leaflets. En face analysis, aortas, including the ascending arch, thoracic, and abdominal segments, were dissected, gently cleaned of the adventitia, and stained with Sudan IV. Exactly the aortic parts were then stretched between two glass plates and fixed in neutral aqueous phosphate-buffered formaldehyde solution. The fixed aortic parts were stained en face with a filtrated solution containing Sudan IV (0.5 g Sudan IV dissolved in 200 ml ethanol∶acetone∶H_2_O, 21∶10∶9, vol/vol/vol; Sigma Chemical, Milan, Italy) to demonstrate areas of developed atheromatous plaques. To identify early atherosclerotic lesions, the aortas were then stained with Sudan IV without removing the pins. Aortas were briefly rinsed in 70% ethanol, immersed for 6 minutes in a filtered solution containing 0–5% Sudan IV (Sigma Chemical Co), 35% ethanol, and 50% acetone for 6 minutes, and destained for 5 minutes in 80% ethanol. The mean lesion area was quantified from ten digitally captured sections per mice using a BX60 microscope (Olympus Co., Rome, Italy) and digitalized using a SPOT-2 camera (Diagnostic Instruments Inc., Sterling Heights, MI) with a resolution of 1315×1033 pixels. Area measurements (expressed in pixels) were done using the free software Image J 1.33u (Rasband W., National Institutes of Health; Bethesda, MD). For liver histology, samples of the right and left liver lobes (100 mg/each) taken from each animal were fixed in 10% formalin, embedded in paraffin, sectioned, and stained with H&E.

### Tissue triacylglycerols and FFA

For determination of total triglyceride, cholesterol and FFA (N = 6-4) content about 100 mg fragments of liver or heart were homogenized with 1 ml of T-PER (Pirce). The homogenized were used for protein concentration, Bradford assay (Bio-rad), and at 100µl of lised tissue extract was added 1.6 ml CHCl3 ∶ MeOH (2∶1) for 16 h at 4°C, after which 200µL of 0.6% NaCl was added and the solution centrifuged at 2,000 g for 20 min. The organic layer was removed and dried by speed vac system (HETO-Holten). The resulting pellet was dissolved in 100µL of phosphate buffered saline containing 1% Triton X-100 and triglyceride and free fatty acids content ware determined using specific enzymatic reagents, the results were expressed for mg of protein.

### Flow cytometry quantification of CD36 expression on monocytes/macrophages

Blood-derived macrophages were collected from wild-type and ApoE−/− mice (n = 12/genotype) as described in the previous section. The cells were treated with ACK solution to eliminate red blood cells, stained by anti-CD14-PE antibody and anti-CD36 antibody (BD Biosciences Pharmingen, San Diego, USA) and analyzed by a flow cytometer (Beckman Coulter, Fullerton, CA).

### Cell Culture and HIV PI Treatment

RAW264.7 cells were obtained from ATCC (Manassas, USA), cultured in RPMI 1640 medium (Sigma) supplemented with 10% fetal bovine serum (Gibco BRL), 100 U penicillin per milliliter, 100 µg of streptomycin per milliliter, and incubated in 10% CO2 in air at 37 8C. Cells were plated on six-well plates (100.000 for well). The medium was replaced after 24 h, and treated. Ritonavir and Atazanavir were dissolved in DMSO. In the first set of experiments HIV PI were directly added to culture medium (final concentration, 5 to 40 µM, DMSO 0.1%) alone for 16 hours at the end of incubation the cells were collected to evaluated cells dead and CD36 expression by flow cytometry. In a second set of experiments the cells were preincubated for 24 h with Genfibrozil (250µM) or CDCA (50µM) and then treated with ritonavir 10 µM for 16 h, at the end of incubation the cells were collected to evaluated CD36 expression by flow cytometry and for mRNA extraction. Total RNA (10 µg) was used for first-strand cDNA synthesis. The mRNA levels of, CD36, SREBP1-2, SHP and PPARγ were determinate by quantitative real-time polymerase chain reaction (qRT-PCR).

### Measurement of Cholesterol up-take

Mouse RAW264.7 macrophages were plated on six-well plates (100.000 for well). The medium was replaced after 24 h, and cells were preincubated for 24 h with Genfibrozil (250µM) or CDCA (50µM) and then treated with ritonavir 10 µM for 16 h, at the end of incubation 10µg/ml of low density lipoprotein acetylated Dil complex (Dil AcLDL) (Molecular Probe, Oregon, USA) was added for 3 hours at 37°. At the end of incubation, cells were collected and washed with PBS and the LDL up-take were measured by flow cytometer (Beckman Coulter, Fullerton, CA).

### Real-time PCR analysis

Quantification of genes expression in tissues was performed by qRT-PCR as described previously [44]. Quantization of the expression of selected genes was performed by quantitative real-time PCR (qRT-PCR). Total RNA were obtained from livers and cells and isolated with TRIzol reagent (Invitrogen), incubated with DNase I and reverse-transcribed with Superscript II (Invitrogen) according to manufacturer specifications. For real-time PCR, 100 ng of template was used in a 25-µl reaction containing a 0.3 µM concentration of each primer and 12.5 µl of 2× SYBR Green PCR Master Mix (Bio-Rad Laboratories, Hercules, CA). All reactions were performed in triplicate using the following cycling conditions: 2 min at 95°C, followed by 50 cycles of 95°C for 10 s and 60°C for 30 s using an iCycler iQ instrument (Bio-Rad Laboratories). The mean value of the replicates for each sample was calculated and expressed as cycle threshold (C_T_). The amount of gene expression was then calculated as the difference (ΔC_T_) between the C_T_ value of the sample for the target gene and the mean C_T_ value of that sample for the endogenous control (GAPDH). Relative expression was calculated as the difference (ΔΔC_T_) between the ΔC_T_ values of the test and control samples for each target gene. The relative level of expression was measured as 2^−ΔΔCT^. All PCR primers were designed using the software PRIMER3-OUTPUT using published sequence data obtained from the NCBI database. Mouse primers were as follows:

mSREBP1c: gatcaaagaggagccagtgc and tagatggtggctgctgagtg;

mSREBP2: acagatgccaagatgcacaa and ttcagcaccatgttctcctg;

mFAS: tgggttctagccagcagagt and accaccagagaccgttatgc;

mHMGCoAS: ggtggatgggaagctgtcta and acatcatcgagggtgaaagg;

mHMGCoAR: ccgaattgtatgtggcactg and ggtgcacgttccttgaagat;

PPARγ: gccagtttcgatccgtagaa and aatccttggccctctgagat;

mFXR: tgtgagggctgcaaaggttt and acatccccatctctctgcac;

mSHP: tctcttcttccgccctatca and aagggcttgctggacagtta;

mCD36: cggagacatgcttattgagaa and actctgtatgtgtaaggacct

mABCA1: agccagaagggagtgtcaga and catgccatctcggtaaacct;

mGAPDH: ctgagtatgtcgtggagtctac and gttggtggtgcaggatgcattg;

m18S: accgcagctaggaataatgga and gcctcagttccgaaaacc.

### Nuclear and cytoplasmic extracts

Nuclear and cytoplasmic extracts from Raw264.7 cells left untreated or administered with 10 µM Ritonavir alone or in combination with 50 µM CDCA or with 250 µM Gemfibrozil were made with the NE-PER kit (Pierce) according to the manufacturer's protocol.

#### Western Blotting

Total lysates from RAW264.7 cells were prepared by solubilization in NuPAGE sample buffer containing Sample reducing agent. Proteins were separated by polyacrylamide gel electrophoresis, transferred to nitrocellulose membranes (Bio-Rad, Hercules, CA) and than probed with primary anti-SREBP-1 antibody (Santa Cruz Biotech, Santa Cruz, CA). The anti-immunoglobulin G Rabbit (Bio-Rad) was used as a secondary antibody, and specific protein bands were visualized by chemoluminescence using Super Signal West FEMTO reagent (Pierce, Rockford, IL).

### Electrophoretic mobility shift assay (EMSA)

Preparation of nuclear extract from RAW264.7 cells was done using NE-PER (Pierce). The probes used for EMSA (CD36-SRE wild type and mutated) were labelled with biotin using Biotin 3′ end DNA labelling kit (Pierce) according to the manufacturer's instructions. For EMSA, 5 µg of nuclear extract from RAW264.7 cells naive or treated with ritonavir alone or in combination with Gemfibrozil or CDCA were incubated with 15 fmol of the CD36-SRE probe, while 5 µg of nuclear extract from stimulated cells was incubated with CD36-SRE-mutated probe in a total volume of 20 µL of binding buffer (50 mmol/L NaCl, 10 mmol/L Tris-HCl, pH 7.9, 0.5 mmol/L EDTA, 10% glycerol, 1 µg of poly dI-dC) for 20 min at room temperature. For competition assays, an excess of CD36-SRE unlabeled oligonucleotides were pre-incubated with nuclear extract from treated cells for 15 min prior to the addition of the biotin-labelled CD36-SRE probe. For antibody-mediated supershift assay, extracts from stimulated cells were pre-incubated with 1 µg anti-SREBP-1 antibody (Santa Cruz Biotechnology, Santa Cruz, CA, USA) at room temperature for 20 min before the addition of the biotin-labelled CD36-SRE probe. The reactions were loaded on a 6% polyacrylamide non-denaturing gel in 0.5× Tris-borate-EDTA buffer and electrophoresed for 1 h at 100 V. The protein/DNA complexes were then transferred to positively charged nylon membrane (Pierce) and the supershift was detected using the Chemiluminescent Nucleic Acid Detection Module (Pierce).

Additional methods are described in “Materials and Methods S1”.

## Supporting Information

Materials and Methods S1 (0.03 MB DOC)

Figure S1 In the absence of ritonavir, administering ApoE^−/−^ mice with an FXR and PPARα agonist reverts dyslipidemia. Gemfibrozil and CDCA reduced plasma levels of total cholesterol, LDL, and triacylglycerols in ApoE^−/−^ mice. In each panel, data are mean± SE of 12 animal per group. *P<0.05 ApoE^−/−^ versus naive; # P<0.05 treatments versus ApoE^−/−^.
(0.31 MB TIF)

Figure S2 HIV protease inhibitors increase CD36 expression on cultured macrophages RAW264.7 cells. Ritonavir and atazanavir, two HIV PIs increases the cell surface expression of CD36 in a concentration-dependent manner. Data are mean ± SE of 4 separate experiments. Natural and synthetic FXR ligands, CDCA and GW4064, counteracts CD36 induction caused by ritonavir and atazanavir. Data are mean± SE of 4 separate experiments.
(0.18 MB TIF)

Figure S3 Nubiscan analysis (http://www.nubiscan.unibas.ch) of the mouse CD36 promoter region revealed one potential SREBP1 binding site, TTCACACCAG, located at −1562 bp from the transcriptional starting site ATG. To assess the functionality of this SRE (Sterol Regulatory Element) we carried a ChIP experiment with an anti SREBP1 antibody (see Materials and Methods S1). As shown, quantitative Real-Time PCR analysis of the CD36 5'flanking region revealed that SREBP1 was unable to bind to the promoter region containing the putative SRE in cells untreated as well as in cells stimulated with ritonavir alone or with the combination ritonavir plus CDCA. These results suggest that SREBP1 regulates CD36 expression with an indirect mechanism.
(0.61 MB TIF)
