# Supplementary material for: The Bile Acid Sensor FXR Protects against Dyslipidemia and Aortic Plaques Development Induced by the HIV Protease Inhibitor Ritonavir in Mice
Source: PLoS One. 2010 Oct 8;5(10):e13238. doi: 10.1371/journal.pone.0013238 (PMC2951893; doi:10.1371/journal.pone.0013238)
Supplement: Materials and Methods S1 — (0.03 MB DOC) [file pone.0013238.s001.doc]

**Materials and Methods S1**

**Chromatin Immunoprecipitation Assay**

Cross-linking and chromatin immunoprecipitation (ChIP) assay was performed according to the manufacturer's protocols (Abcam, Cambridge,  UK) with minor modifications.  Briefly, RAW 264.7  cells were  cross-linked with 1% formaldehyde at room temperature and then the  reaction terminated by the addition of glycine  at a final 
concentration of 0.125 M. Cells were washed in ice-cold PBS and lysed  with SDS lysis buffer (1% SDS, 10 mM EDTA, and 50 mM Tris-HCl, pH 8).  Cellular lysates were diluted with ChIP dilution buffer, sonicated,  and immunoprecipitated with specific antibodie anti-SREBP-1 from  (Santa Cruz Biotech, Santa Cruz,  CA). Immunoprecipitates were  collected with protein A beads (Amersham Bioscience) and washed 
sequentially first with a low-salt wash buffer and then with high-salt  wash buffer using manufacturer's recommended procedures. DNA was  eluted by addition of 1% SDS and 0.1 M NaHCO3, and the cross-linking  reactions were reversed by heating the mixture to 65°C overnight. The  DNA was recovered from immunoprecipitated material by proteinase K  treatment at 65°C for 1 h followed by phenol/chloroform (1:1) 
extraction, ethanol precipitation and dissolved into 50 µl of water.   For Real-Time PCR,  5 µl of ChIP products  from RAW264.7 were used to  perform amplification of mouse CD36 gene promoter using the following  primers against the SREBP-1 binding site: TTCACACCAG.
